# Drug–Drug Compatibility Evaluation of Sulfasalazine and Folic Acid for Fixed-Dose Combination Development Using Various Analytical Tools

**DOI:** 10.3390/pharmaceutics13030400

**Published:** 2021-03-17

**Authors:** Mario-Livio Jeličić, Edvin Brusač, Stanislav Kurajica, Matija Cvetnić, Daniela Amidžić Klarić, Biljana Nigović, Ana Mornar

**Affiliations:** 1 Faculty of Pharmacy and Biochemistry, University of Zagreb, A. Kovačića 1, 10000 Zagreb, Croatia; mljelicic@pharma.hr (M.-L.J.); ebrusac@pharma.hr (E.B.); damidzic@pharma.hr (D.A.K.); bnigovic@pharma.hr (B.N.); 2 Faculty of Chemical Engineering and Technology, University of Zagreb, Marulić Square 19, 10000 Zagreb, Croatia; stankok@fkit.hr (S.K.); mcvetnic@fkit.hr (M.C.)

**Keywords:** sulfasalazine, folic acid, fixed-dose combination, physicochemical compatibility, thermal techniques, spectroscopy, chromatography

## Abstract

The simultaneous administration of sulfasalazine and folic acid is regular practice in the therapy of inflammatory bowel diseases in order to maintain sufficient folate concentration in patients. Having multiple drugs in the therapy increases the possibility of patients failing adherence, thus unintentionally endangering their health. A fixed-dose combination of sulfasalazine and folic would simplify the classical polytherapeutic approach; however, the physicochemical compatibility investigation of two active pharmaceutical ingredients plays an important role in the development of such a product. In this work, various analytical tools were used to determine the physicochemical compatibility of sulfasalazine and folic acid. For the evaluation of chemical compatibility, infrared spectroscopy in combination with advanced statistical methods, such as the principal component analysis and cluster analysis, were used, whilst a simultaneous thermogravimetry/differential thermal analysis gave us an insight into the physical compatibility of two drugs. Isothermal stress testing, forced degradation and dissolution studies, followed by the analysis with a developed chromatographic method for the monitoring of folic acid, sulfasalazine and two of its related impurities, sulfapyridine and salicylic acid, gave us an insight into its chemical compatibility. The combination of the results obtained from the used techniques implies a satisfactory physicochemical compatibility between sulfasalazine and folic acid, which opens the path to the development of the proposed fixed-dose combination.

## 1. Introduction

Sulfasalazine (SASP) is an anti-inflammatory drug that has been mainly used for the treatment of rheumatoid arthritis since its discovery in the 1930s. Nevertheless, patients with ulcerative colitis given the SASP therapy have noticed a significant reduction of symptoms, which ultimately led to its use in the treatment of inflammatory bowel diseases (IBD) [1]. The SASP molecule consists of the anti-inflammatory 5-aminosalicylic acid and an antibiotic sulfapyridine (SP) connected via an azo bond. Once administered, SASP is absorbed up to 25% in the small intestine, while the rest of the drug reaches the colon mostly unchanged, where enzyme azo-reductase breaks the azo bond, releasing 5-aminosalicylic acid at the site of inflammation [2]. The main downsides of SASP are the toxicity of its metabolite SP, which is responsible for its side effects in nearly 30% of patients, and the SASP-induced inhibition of the reduced folate carrier at clinically relevant plasma concentrations. This inhibition leads to a folate deficiency in patients that consequently increases the chances of developing colorectal cancer and megaloblastic anemia, as well as defects, in newborns [3,4,5,6]. To maintain a sufficient folate concentration in IBD patients, a supplementation of folic acid (FA) during the regular therapy of IBD is mandatory, usually in elevated doses of 1–5 mg/day compared to the intake of 0.4 mg/day in healthy people [7].

A fixed-dose combination (FDC), a drug formulation comprising of multiple active pharmaceutical ingredients (APIs), is a great alternative to the classical therapy approach. For diseases that require polytherapy, such as IBD, multiple drugs must be administered simultaneously, increasing the risk that some of the medications will be omitted. SASP/FA FDC would increase the medication adherence in IBD patients by ensuring the regular intake of FA, where the proposed FDC would contain enough SASP and FA to maintain the remission state of the disease and to supplement malabsorbed FA during the therapy. The process of FDC development requires thorough drug–drug compatibility testing to ensure the physical and chemical compatibility of APIs. Physical and chemical incompatibility can result in unwanted interactions between two drugs leading to the questionable safety, efficacy and stability of the final product. Physical incompatibility can result in the change of odor, color and morphology whilst chemical incompatibility can lead to the creation of new inactive or toxic products [8]. Various techniques and methods, such as Fourier-transform infrared spectroscopy (FTIR), simultaneous thermogravimetry, differential thermal analysis (TG/DTA) and isothermal stress testing (IST), have been used so far for examining drug–drug and drug–excipient physicochemical compatibility [9,10,11,12]. Forced degradation studies, comprising of a series of degradation conditions applied to APIs and finished products, as well as their blends, are recommended during the FDC development process by The World Health Organisation guidelines for the registration of FDCs [13].

**Figure 1:**
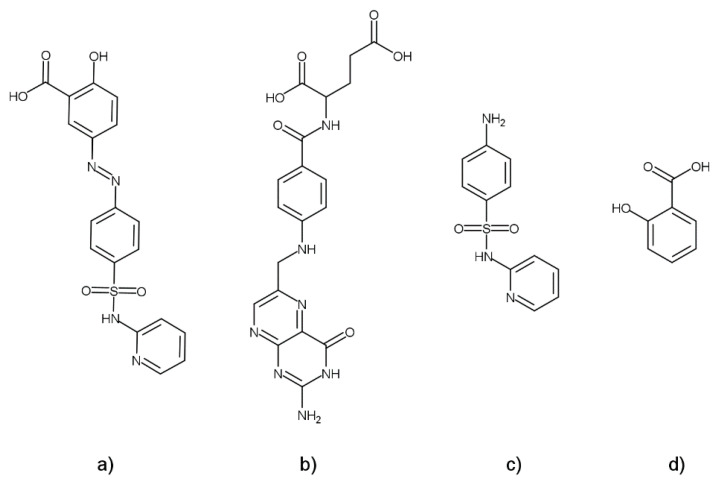
Structures of (**a**) sulfasalazine (SASP), (**b**) folic acid (FA), (**c**) sulfapyridine (SP) and (**d**) salicylic acid (SA).

Up until now, there has been no published study concerning the physicochemical compatibility investigation of SASP and FA when present in the same solution or blend. In this research, the physicochemical compatibility of SASP and FA was examined using the FTIR, TG/DTA and IST techniques, as well as forced degradation and dissolution studies. For the assay determination after the IST, forced degradations and dissolution studies, a single high-performance liquid chromatography (HPLC) assay method was developed for the monitoring of SASP, FA and two SASP-related impurities, SP and salicylic acid (SA) (Figure 1).

## 2. Materials and Methods

### 2.1. Reagents and Chemicals

The SASP (as per the British Pharmacopoeia (BP), 99.6%), FA (meets the United States Pharmacopoeia (USP) specifications, 100.6%), SP (98.0%) and SA (≥99.0%) standards were provided by Sigma-Aldrich (St. Louis, MO, USA). Chemicals used for the preparation of samples, buffers, mobile phase, pH adjustment and degradation studies were the following: ultrapure water (<0.1 µS/cm) purified with a Series Ultra Clear and Integra Ultra-pure water system (SG Water, Barsbuttel, Germany), methanol and acetonitrile HPLC grade solvents (≥99.9%) (Avantor, Deventer, The Netherlands), *N*,*N*-dimethylformamide (DMF) (99.5%) (VWR Chemicals, Radnor, PA, USA), formic acid for HPLC (98–100%) (Merck, Kenilworth, NJ, USA), glacial acetic acid (99.5–100.5%) (Panreac Química S.L.U., Barcelona, Spain), ammonium acetate (≥98.0%) (Lach-Ner s.r.o., Neratovice, Czech Republic), sodium hydroxide pellets (≥98.0%) (Sigma-Aldrich), hydrochloric acid (37.0%) (Carlo Erba, Val-de-Reuil, France), hydrogen peroxide (≥30.0%) (T.T.T. d.o.o., Sveta Nedelja, Croatia), monobasic potassium phosphate (≥99.0%) (Kemika, Zagreb, Croatia) and α-alumina (Sigma-Aldrich).

Finished drug products Sulfasalazin Krka EN 500-mg gastro-resistant tablets (KRKA, d.d., Novo Mesto, Slovenia) and Folacin^®^ 5-mg tablets (Jadran-Galenski Laboratorij d.d., Rijeka, Croatia) used in experiments were obtained from the local pharmacy. For selectivity tests and method validation, the following excipients (present in the above-stated drug products) were used for the preparation of the placebo blend: lactose monohydrate, cellulose (microcrystalline), povidone, crospovidone, starch, magnesium stearate and silicon dioxide (colloidal anhydrous). All excipients were obtained from Fagron Hrvatska d.o.o. (Donja Zelina, Croatia).

### 2.2. Preparation of Working Solutions and Blends

Stock solution containing 1000-µg/mL SASP, 2-µg/mL FA, 5-µg/mL SP and 5-µg/mL SA was prepared by carefully weighing and dissolving each standard in 30% DMF. Working solution, containing 250-µg/mL SASP, 0.50-µg/mL FA, 1.25-µg/mL SP and 1.25-µg/mL SA, was prepared by diluting the stock solution with 30% DMF and the addition of the placebo blend followed by 10-min sonication in an ultrasonic bath (Elmasonic XtraTT, Biosan, Riga, Latvia). Working solution contained the maximum allowed concentration of impurities (in relation to SASP), as per the BP monograph for the SASP finished product [14]. Prior to HPLC analysis, the working solution was centrifuged and filtered through a polyethersulfone (PES) syringe filter (25 mm, 0.22 µm) (Obrnuta faza d.o.o., Pazin, Croatia) to remove the residues of undissolved excipients.

Standard and tablet blends for IST tests and forced degradation studies were prepared in the proposed 500:1 ratio by weighing (using a MX5 microbalance scale by Mettler Toledo, Columbus, OH, USA) and mixing 2000 mg of SASP and 4 mg of FA standard or, in the case of tablet blend preparation, by weighing the amount of previously powdered tablets equivalent to 2000 mg of SASP and 4 mg of FA. Standard blends for TG/DTA measurements were prepared in a 1:1 ratio by mixing equal amounts of each standard, whilst, for the FTIR measurements, 500:1, 10:1, 5:1, 2:1, 1:1, 1:2, 1:5 and 1:10 blends were prepared. Placebo blend was prepared by mixing the commonly used excipients in a ratio that represented their maximum allowed concentration in the formulation [15]. All powders were thoroughly mixed using mortar and pestle for 20 min to secure complete homogenization.

### 2.3. Chromatographic Conditions

Agilent 1100 series HPLC system (Agilent Technologies, Waldbronn, Germany) coupled with a diode array detector with Chemstation software for data processing was used for all chromatographic analyses. Separation was performed on XBridge C18 (150 × 4.6 mm, 3.5-µm particle size) reverse-phase column with suitable guard column, both obtained by Waters (Milford, MA, USA). The column was thermostated at 25.0 ± 0.1 °C during the analysis, with a constant flow rate of 1.0 mL/min. The mobile phase consisted of 5.0-mM ammonium acetate buffer (pH set to 5.00 ± 0.01 using a FiveEasy pH meter by Mettler Toledo) as mobile phase A and methanol as mobile phase B. Gradient elution program was as follows: 0–8 min, linear gradient 5–40% B; 8–10 min, linear gradient 40–80% B; 10–15 min, isocratic 80% B and 15–18 min, linear gradient 80–100% B. Analysis time was 18 min, with an additional 7 min to re-equilibrate the stationary phase for the following analysis. Injection volume of 5.0 µL and detection wavelength of 360 nm was chosen for the monitoring of SASP, whilst FA, SA and SP were monitored at 285 nm with a 20.0-µL injection volume.

### 2.4. TG/DTA Analyses, IST Conditions and FTIR Settings

TG/DTA analyses were carried out using the NETZSCH thermal analyser STA 409 (NETZSCH-Gerätebau GmbH, Selb, Germany). Approximately 10.0 ± 0.1 mg of the sample was carefully weighed directly into the priorly cleaned platinum pan. Measurements were performed under the atmosphere of synthetic air in a flow rate of 30 mL/min from 25 °C up to 400 °C, with the heating rate of 10 °C/min and the usage of α-alumina as a reference.

IST was performed on SASP and FA standards, tablets, standard blends and tablet blends. Samples were spread in a thin layer on a Petri dish, visually inspected and placed in the thermostat (ES-20/60 Orbital Shaker-Incubator by Biosan) for 4 weeks at a temperature of 50 °C. After the heating period, samples were examined to identify any visual changes and analyzed using the developed HPLC method and FTIR.

For the chromatographic analysis, 5 mg of stressed sample was weighed and dissolved in 20 mL of 30% DMF, centrifuged, filtered and analyzed using the developed method.

Infrared spectra of SASP and FA, as well as all prepared blends, were obtained in the range from 500–4000 cm^−1^ using a Fourier-transform infrared spectrophotometer FTIR-8400S by Shimadzu (Kyoto, Japan) with PIKE MIRacle™ universal ATR by PIKE Technologies (Madison, WI, USA). All spectra were obtained as the average of 45 scans with a resolution of 2 cm^−1^.

### 2.5. Forced Degradation Study Conditions

Forced degradation studies were performed on SASP and FA standards, standards blend and tablets blend. Acid, alkali and oxidative degradation studies were conducted on solutions in 0.1-M HCl, 0.1-M NaOH and 3% H_2_O_2_. Thermal stability of the solutions and solid samples was examined at a temperature of 70 °C, whilst photostability studies were performed by keeping the samples in indirect daylight. Degradation studies were conducted on solutions containing 250 µg/mL of SASP and 0.5 µg/mL of FA, whilst degradations conducted on solids were performed on accurately weighed amounts of prepared samples that were, after the reaction period, dissolved in 30% DMF. Samples were kept in described conditions until the optimal degradation of 10–30% [13]. All samples were analyzed using the developed HPLC method.

### 2.6. In Vitro Dissolution Studies

Dissolution studies of the SASP and FA finished products, as well as their simoultaenous dissolution, were performed as per the USP method for dissolution of SASP delayed-release tablets [16]. The USP 2 dissolution apparatus LDLT-A10 (Labtron Equipment Ltd., Fleet, UK) was used with the paddle rotation speed fixed at 100 rpm and bath thermostated at 37.0 °C. Simulated gastric fluid (SGF; 0.1-N HCl) and simulated intestinal fluid (SIF; 0.50-mM phosphate buffer (PB) solution, pH 7.5) were used as dissolution media at a constant volume of 900 mL for a two-stage dissolution procedure. The first stage involved the dissolution of tablets in SGF for 120 min, followed by 60-min dissolution in SIF. During the test, an aliquot of 5 mL was taken from the dissolution vessel every 30 min for assay determination using the developed HPLC method. An equal amount of fresh dissolution media was added after each sampling to keep the dissolution media volume constant. Dissolution experiments were conducted in triplicate for each tablet and their combination.

### 2.7. Statistical Methods

Statistical software XLSTAT by Addinsoft (New York, NY, USA) was used for interpretation of the obtained FTIR spectra and comparison of the results obtained from the chromatographic analysis of samples conducted for IST.

Statistical data processing was performed using the principal component analysis (PCA) with Pearson’s correlation and a cluster analysis (CA) using Euclidean distance measures and Ward’s agglomerative clustering on the spectral region from 550–1800 cm^−1^, where most of the information was present. A 10 × 650 matrix was created where the number of rows represented SASP, FA and eight of their blends, whilst the columns represented the spectral data obtained with the 2-cm^−1^ resolution.

Two sample *t*-test was used for the comparison of recoveries obtained from the chromatographic analysis of the samples conducted for IST. The first group represented the recoveries of the samples stressed alone, whilst the second were recoveries from the stressed blends.

## 3. Results and Discussion

### 3.1. HPLC Method Development and Validation

**Figure 2:**
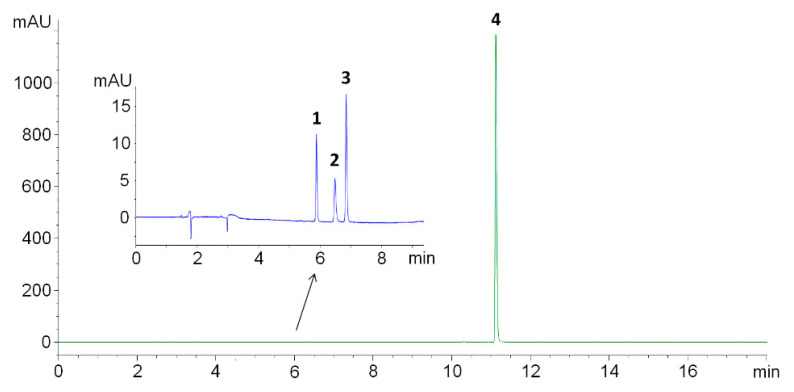
Chromatogram of a working solution showing FA (1), SA (2) and SP (3) at 285 nm (blue line) and SASP (4) at 360 nm (green line).

The development of the stability-indicating assay method started with the selection of the optimal HPLC column. An XBridge C18 reverse-phase column (150 × 4.6 mm, 3.5-µm particle size) with a suitable guard column was used as a starting point for the method development. SASP was expected to be well-retained on the C18 column due to its high log*K*_ow_ value (3.7–4.8) [17]. Using isocratic elution with 50% methanol acidified with 0.1% formic acid as the mobile phase resulted in poor resolution between FA, SP and SA (<1.3), whilst SASP was well-retained, however, with prominent peak tailing (3.8). Considering the poor chromatographic performance obtained with the isocratic elution in acidified conditions (pH 2.87), the mobile phase was replaced with 5.0-mM ammonium acetate buffer with the pH set to 5.00 ± 0.01 with acetic acid. A gradient elution was necessary to get acceptable SASP retention and, thus, overall method run time. Usage of the above-defined conditions resulted in the satisfactory separation of all compounds (resolution > 3.98), peak symmetry (<1.26, as per USP) and purity factor (>998) (Figure 2).

The column temperature and flow rate had a minor impact on the separation and were set to be constant at 25.0 °C and 1.0 mL/min. To increase the sensitivity of the method, FA, SA and SP were monitored at 285 nm with a 20.0-µL injection volume, whilst SASP was monitored at 360 nm with a 5.0-µL injection volume to avoid a column and detector overload.

The method was validated regarding selectivity, linearity, accuracy, precision, limit of quantification, limit of detection and robustness as per the ICH guideline Q2 (R1) [18]. The selectivity of the method was determined using Chemstation software to calculate the peak purity of each peak with the purity factor threshold set at 995. No interferences were observed at the elution time of the analytes, whilst all peaks were shown to be pure, with the purity factor values greater than 998. The resolution factor between all the peaks was higher than 3.98, which exceeds the regulatory proposed minimal value of 1.5.

Five-point calibration curves of high linearity (*r* ≥ 0.999) were obtained in the range from 80% up to 120% of the working solution concentration for SASP and FA and from the limit of quantification up to 180% of the maximum allowed impurity concentration for SA and SP as per the BP [14]. The limits of detection and quantification were determined as 3:1 and 10:1 signal-to-noise ratios, respectively (Table 1).

Six individually prepared samples were analyzed within one day for determination of the intra-day precision, whilst for the inter-day precision, samples in triplicate were prepared and analyzed each day for three consecutive days. The low relative standard deviation (RSD) values of the intra-day (≥0.77%) and inter-day (≥0.78%) precisions implied that the sample preparation process and method remained precise within and between the days. The method accuracy at the three concentration levels was tested in triplicate and expressed as the recovery with accompanying RSD values. The accuracy for SASP and FA was tested in triplicate at 80%, 100% and 120% concentration levels of the working solution, whilst for SA and SP, the accuracy was tested at the limit of quantification as the lowest point, as well as at 100% and 180% of the working solution concentration. Satisfactory results were obtained for all analytes, ranging from 99.14% ± 0.16% up to 103.84% ± 0.76%. The accuracy and precision data are shown in Table 1.

The method was shown to be robust regarding the recovery (assays remained within the regulatory limit of 100.0% ± 5.0%), retention times (RSD < 5%) and resolution (*R*_s_ > 1.5) on the changes in the flow rate (1.00 ± 0.05 mL/min), column temperature (25.0 ± 1.0 °C), mobile phase pH (5.0 ± 0.1) and changes in the gradient (±1% of methanol).

### 3.2. TG/DTA Measurements

**Figure 3:**
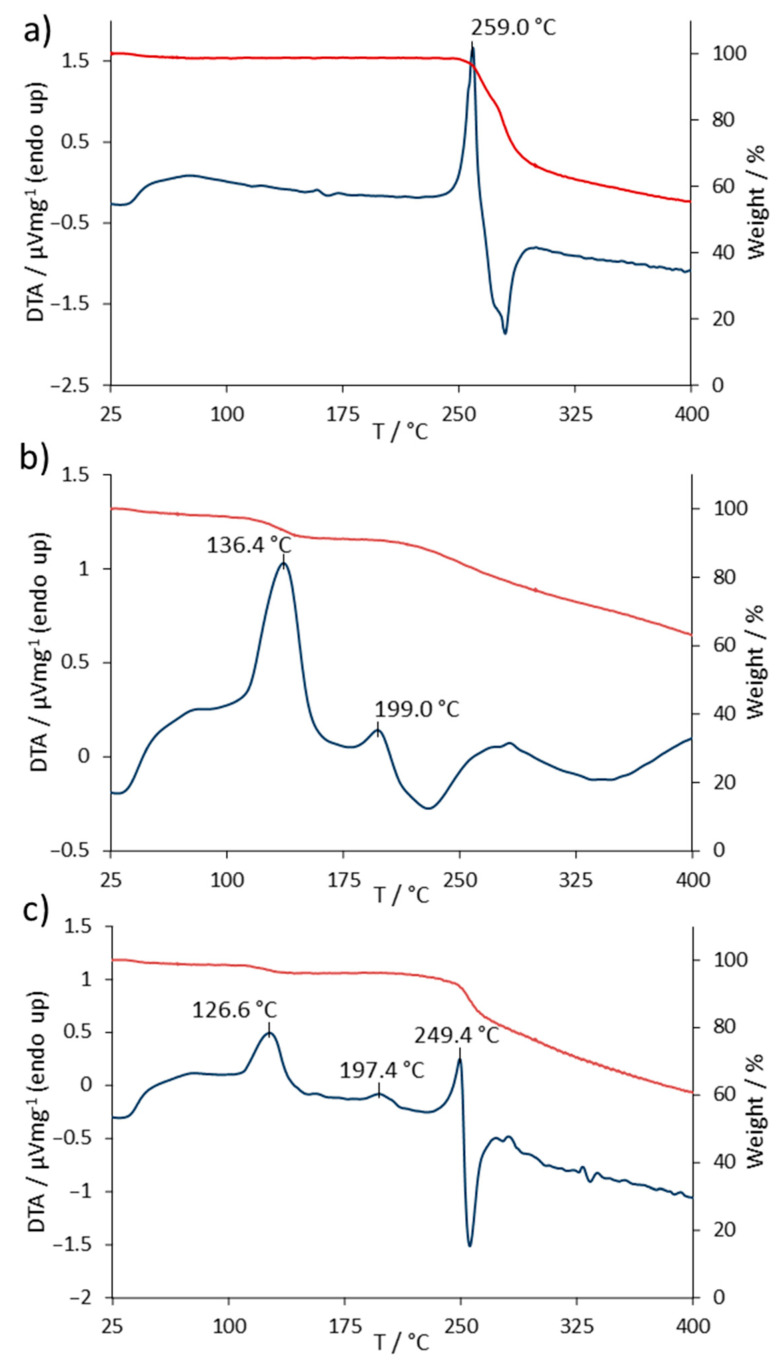
Thermogravimetry (TG) (red line) and differential thermal analysis (DTA) (blue line) curves of (**a**) SASP, (**b**) FA and (**c**) SASP/FA 1:1 blend.

The TG/DTA measurements of SASP and FA were done on a 1:1 binary mixture to increase the intensity of the possible reactions and for a better visualization of the incompatibility [19]. The TG/DTA curves of SASP, FA and the SASP/FA blend are shown in Figure 3. The DTA measurements of SASP showed an endothermic peak at 259.0 °C, which corresponded to its melting point, followed by degradation, which was observed as an exothermic peak. The TG measurement of SASP showed a weight loss of 2.0% in the temperature range of 0–100 °C due to the water evaporation from the sample. The weight remained constant until the beginning of the melting of SASP, followed by its degradation, resulting in a notable loss of 31.9% in the range of 240–300 °C. With the further heating of the sample, a constant weight loss was observed in the temperature range of 300–400 °C, with the final weight of the sample being 55.3% of the starting value (Figure 3a).

The DTA measurements of FA showed two endothermic peaks at 136.4 and 199.0 °C. The peak at 136.4 °C corresponded to the endothermic nature of the water evaporation process. The TG measurements in the range of 0–175 °C showed a weight loss of 8.4%, which suited the declared value of the water content in the used FA standard of 8.0% [20]. The endothermic peak at 199.0 °C corresponded to the FA melting temperature, followed by a slow degradation as the heating continued, which could be seen as a constant loss of weight in the range of 200–400 °C [12]. The weight of the FA sample upon the end of measurements was reduced to 63% of its starting weight (Figure 3b).

The TG/DTA measurements of the SASP/FA blend showed both SASP and FA previously observed characteristic peaks. The endothermic peak at 126 °C corresponded to the loss of water present mainly in the FA standard. A weight loss of 3.9% was observed, which was equivalent to that of 8.4% observed in the FA standard alone, since the SASP/FA blend was prepared in a 1:1 ratio. The FA characteristic melting temperature was observed at 197.4 °C, whilst a slight shift in the SASP endothermic peak occurred, resulting in a temperature of 249.4 °C. The final weight of the sample was 60.8% of its starting weight, which fell in between the observed final weights of the SASP and FA standards, implying that no additional loss in combination was observed (Figure 3c).

The incompatibility of two drugs can result in the change of their physical characteristics, which can be observed as a shift in the characteristic peak temperature (±5 °C) or if the peak notably changes its shape, disappears or a new peak occurs [12,19,21,22]. In the provided measurements, a shift of the peak temperature was observed for SASP (9.6 °C); on the other hand, the FA characteristic melting peak did not shift notably (1.6 °C), which implies that the two drugs might be compatible. Even though the peak shapes remained intact and there was no occurrence of new peaks, the shift in the SASP peak was above the tolerance described in the literature, which might indicate possible changes in the system. However, it is well-known that conclusions cannot be made based only on TG/DTA measurements, since they provide limited information, and the use of other complementary analytical methods is mandatory to get a better understanding of the possible interactions [11,23].

### 3.3. IST Followed by FTIR and HPLC Measurements

**Figure 4:**
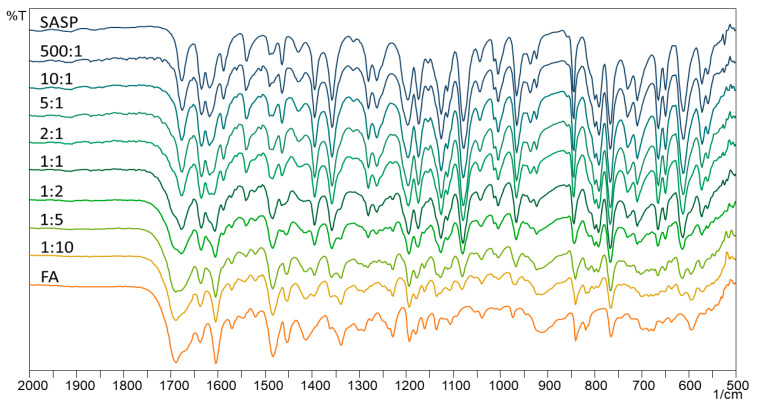
Fourier-transform infrared (FTIR) spectra of SASP, FA and their blends.

In isothermal stress testing, the samples are subjected to isothermal conditions for a longer period, usually two–six weeks, to get an insight into the possible interactions between two or more components in the blend. FTIR is commonly used for getting an insight into the possible chemical changes in the sample, indicating the creation of new bonds, which ultimately results in different FTIR spectra. The FTIR spectra of SASP, FA and their blends are shown in Figure 4.

The obtained spectra show most of the SASP and FA characteristic absorption bands; however, since SASP and FA are molecules with numerous bonds, the FTIR spectra of the physical mixtures are hard to interpret. The SASP characteristic bands (C=O stretching from the carboxyl group at 1674 cm^−1^, S=O from sulphonamide at 1356 cm^−1^ and C–O stretching at 1078 cm^−1^) are more pronounced in the 500:1, 10:1, 5:1 and 2:1 blends; however, as the ratio of SASP and FA equalized (1:1), the FA characteristic bands (C=O stretching at 1687 cm^−1^ and N–H bending at 1602 cm^−1^) appeared and became more dominant as the FA ratio in the blends increased (1:2, 1:5 and 1:10).

The interpretation of the FTIR spectra of the physical mixtures may lead to false conclusions due to the presence of the overlapping bands. Therefore, without going into a deeper visual analysis of the obtained spectra, where, as said above, we cannot gain an unambiguous conclusion, we decided to introduce a statistical approach with the aim of a better interpretation of the obtained spectra. Multivariate statistical analysis tools, such as PCA and CA, were already used and are well-described in the literature for the interpretation of obtained data [24,25].

The results of the PCA analysis showed that eigenvalues of the nine obtained eigenvectors ranged from 494.6 to 0.2, whilst the data variability was in range from 76.21% to 0.03% (Table 2).

The eigenvector with the greatest variability and eigenvalue was the most impactful principal component, implying that the eigenvector with the eigenvalue of 494.6 and a variability of 76.21% was the first principal component (PC1). Since 96.98% of the total variance was covered by the first two eigenvectors (PC1 and PC2), the eigenvalues and variabilities of the last seven eigenvectors (three–nine) were insignificant and, therefore, were excluded from further analyses.

**Figure 5:**
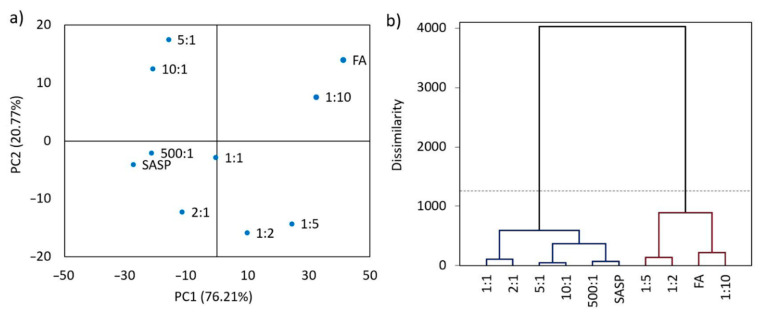
Results of the statistical analysis showing (**a**) the principle component analysis (PCA) bidimensional scatterplot and (**b**) cluster analysis (CA) dendrogram, indicating similarities between SASP, FA and their blends.

For a better visualization of the obtained principal components and data variability, the PCA results were best visualized using a PC1 vs. PC2 bidimensional scatterplot (Figure 5).

FA is located far to the right with the most positive PC1 value (41.33), whilst SASP is located far to the left with the most negative PC1 value (−27.49), where the blends of SASP and FA are placed in an increasing PC1 order as the SASP ratio in the blends reduces and the FA ratio increases (Figure 5a). These results are in favor of the SASP and FA chemical compatibility, showing the similarity between the spectra of the two adjacent blends. Random placement of the samples along the PC1 axis would indicate that there is no similarity between the obtained spectra due to the possible chemical reaction between the components in the blends.

The cluster analysis (Figure 5b) precisely reflected the results present in the scatterplot. Two main clusters were formed; the first connected SASP and the blends with a higher SASP share (500:1, 10:1, 5:1 and 2:1), whilst the other connected FA and its blends with a higher FA share (1:10, 1:5 and 1:2). The SASP/FA 1:1 blend was placed in a cluster with stronger linkage to the SASP-dominant blends, since the SASP spectrum overlapped most of the FA characteristic bands. The dissimilarity values between the formed clusters were below the strong Sokai and Sneath distance criteria (33% of the maximum distance, dashed line), which indicated that there was a statistically significant similarity between the obtained spectra, therefore implying that no chemical reaction occurred in the blends.

The IST is usually followed by HPLC measurements to determine any assay drops as a result of drug–drug interactions. Table 3 shows the results of the chromatographic analysis, as well as the visual inspection observations.

The HPLC analysis results showed a great stability under the imposed conditions, with little to no change in the determined assays. With the statistical analysis, using the two-sample *t*-test, it was determined that the recovered assays were not significantly different (*t*-value = 0.72, *t*-critical = 3.18, *p*-value = 0.52, *α* < 0.05) in the stressed blends from those when stressed alone.

### 3.4. Forced Degradation Study

Forced degradation studies were applied on SASP and FA standards, standards blend and tablets blend to get an insight into the degradation behavior of each component when stressed alone and in the presence of each other. The goal of this approach was to examine the chemical stability of SASP and FA and to determine whether they impacted each other’s stability in the applied conditions. Samples were exposed to alkali, acid, oxidative, thermal and photolytic conditions and kept in the same until the optimal degradation of 10–30%. Keeping the samples too long in defined conditions would result in extensive degradation and the formation of unwanted secondary or tertiary degradation products, which could misslead the experiment; however, if the analyte shows little to no degradation in the seven-day period, it can be stated that the substance is stable in the applied conditions. The optimal degradation time used for stressing of the blends was the time needed for the first standard to degrade in the desired range when stressed individually; therefore, using that same reaction time for both blends’ comparable results can be obtained regarding the degradation of that component. Stressed samples were analyzed using the developed HPLC method to determine the assay drops, as well as occurrence of SASP-related impurities of SA and SP. Degradation times and assay losses of stressed samples are presented in Table 4.

As can be seen from the results, the SASP standard remained stable in all conditions for 7 days, with the maximum assay drop of 1.9% observed in thermally stressed solution. FA solution was shown to be stable in alkali and thermal conditions for 7 days with the assay drop not exceeding 2.7%. Solid samples of FA exposed to thermal and photolytic conditions for 7 days measured assay drop of not more than 2.9%, implying stability in those conditions. FA solution was shown to undergo degradation in acid, peroxide and photolytic conditions, after 1 day, resulting in assay drops of 21.6%, 11.7% and 10.0%, respectively. SASP remained stable when combined with FA in prepared standard and tablet blends through 7 (or 1) day exposure to all conditions, with the maximum degradation of 2.4%. FA in blends has shown similar stability in all stress conditions except when heat-stressed as standard and tablet blend solution, where severe degradation occurred with FA assay drop of 100% after 1 day, compared to 1.1% degradation after 7 days as standard solution. Considering that FA is highly unstable in low pH environment, especially at elevated temperatures [26], as it was observed in our study (17.4–21.6% assay drops at room temperature and 100% at 70 °C), this phenomena can be justified with the fact that prepared 250 µg/mL SASP solution in 30% DMF measures pH of 2.85 (compared to pure 30% DMF solution that measures pH of 6.35), creating the acidic environment in which FA undergoes degradation, whilst heating of the sample significantly increases reaction kinetics, which ultimately resulted in complete FA degradation in a short period. Considering the absence of SASP/FA interactions observed with the previous methods, it can be said that the observed drop in FA assay is strictly pH & temperature related, rather than as a result of chemical interaction between SASP and FA. The impact of pH on instability of FA as well as the impact of pH in combination with elevated temperatures is well described in the literature [27,28,29,30]. However, given that SASP is responsible for creating a low pH environment when dissolved, this must be taken into consideration during the formulation process. In all degradations conducted SASP related impurities SP and SA were not detected. Purity of SASP and FA chromatographic peaks remained higher than 998 after every degradation conducted (purity threshold set to 995 whilst purity value of 1000 represents the identical spectra).

### 3.5. Drug Dissolution Testing

The impact of drugs on their release and stability can be examined with in-vitro dissolution studies using simulated gastric and intestinal fluids [10,12,31,32]. Drug release and stability were tested according to USP procedure using the two-stage dissolution process. The first stage concerned 120-min dissolution of tablets in 0.1 N HCl solution, also known as USP simulated gastric fluid. The second stage involved 60-min dissolution in 0.05 mM PB with pH set to 7.5, also known as USP simulated intestinal fluid. A two-stage dissolution process is necessary since the tested SASP product comes in a form of gastro-resistant tablets, securing that most of the drug is released in the colon, rather than in the stomach.

**Figure 6:**
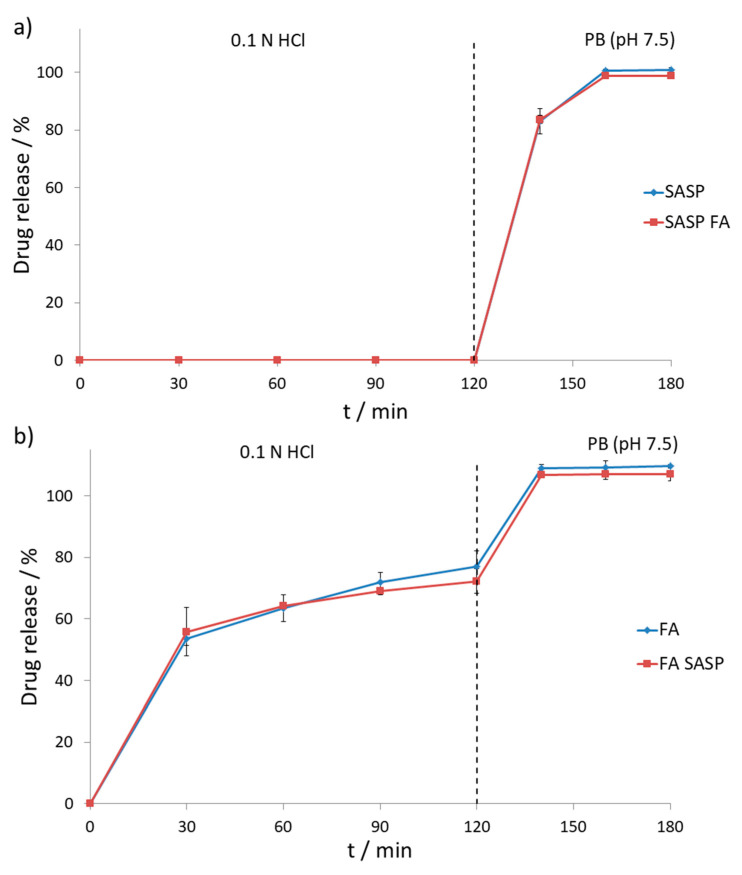
Drug release profiles of (**a**) SASP and (**b**) FA alone (blue line) and in combination (red line) in different dissolution media (*n* = 3) (Dashed line represents the change of dissolution media).

As expected, SASP was not released from the formulation during the first two hours of the first dissolution stage, followed by the second stage with the gradual release of SASP over one hour resulting in a total release of 100.7% ± 0.8%. The effect of FA on the SASP release was tested by placing the SASP and FA tablets in the dissolution vessel at the beginning of the experiment to test the impact of dissolved FA formulation on the gastro-resistant film of the SASP tablet, which could result in premature release of SASP from the formulation. In the two-hour acid stage, no SASP was released from the formulation whilst in the following stage release of 98.8% ± 0.4% was observed. Both SASP alone and SASP in the presence of FA gave similar release curves (Figure 6a), which can be confirmed by calculating the difference (*f*_1_) and similarity factors (*f*_2_) [33]. The calculated difference factor of 1.6% is well below the maximum limit of 15.0%, whilst a high similarity factor value of 92.4% implies great similarity, considering that the 100.0% similarity value corresponds to two identical curves.

On the other hand, FA showed a gradual release in simulated gastric fluid, resulting in the recovery of 77.1% ± 4.9% during the first two hours, followed by the release of 109.7% ± 0.6% in the second dissolution stage. The possible occurrence of incompatibility between FA and SASP, considering the previously observed degradation in the forced degradation study, was tested by placing both tablets in the dissolution vessel at the beginning of the experiment. In both the acid and buffer stages, FA showed similar release as when tested alone, with the recovery values of 72.3% ± 4.0% and 107.2% ± 2.2%, respectively (Figure 6b). Difference (*f*_1_) and similarity factors (*f*_2_) were calculated as well with obtained values of 3.01 and 77.91, respectively, implying that the obtained curves were similar, and no significant interaction between two drugs occurred.

## 4. Conclusions

SASP and FA physicochemical compatibility was successfully examined using various analytical techniques. DTA measurements showed a shift of the SASP peak when present in a blend with FA; however, no changes of the FA peak and no occurrence of new peaks nor the disappearance of existing peaks were observed. The TG measurements showed no significant changes in weight loss, which indicated a lack of interactions; however, conclusions cannot be made solely on the TG/DTA measurements. The FTIR measurements in combination with the PCA/CA analysis implied that no interaction occurred between SASP and FA, which was also confirmed with the chromatographic analysis of the samples after IST, forced degradation and dissolution studies. Considering the ratio in which the SASP and FA samples were prepared (500:1), a significant loss of the assay of FA could be observed if any chemical interaction occurred. The thermal stress study of the SASP/FA standard and tablet solution resulted in the complete degradation of FA; however, it was explained as a pH- and temperature-related process and not a result of the SASP and FA interaction. Although the TG/DTA measurements raised concerns, with the wider picture obtained using various analytical tools, this work implies that SASP and FA are physicochemically compatible. This work presents one segment of the FDC development process; however, further studies, such as selecting optimal excipients, drug–excipient compatibility studies and formulation stability studies, as well as dissolution studies of prepared dosage units, should be conducted to create an acceptable product.

## Figures and Tables

**Table 1:** Method validation data.

**Analyte**	**Relative Retention Time**	**Linearity Range (µg/mL)**	**Regression Equation**		***r***	**Limit of Detection (µg/mL)**		**Limit of Quantification (µg/mL)**
SASP	1.00	200–300	*y* = 15.12 *x* + 373.40		0.9999	/		/
FA	0.53	0.4–0.6	*y* = 64.79 *x* − 0.04		0.9999	0.03		0.10
SA	0.58	0.13–2.25	*y* = 19.02 *x* + 0.14		0.9997	0.04		0.13
SP	0.62	0.13–2.25	*y* = 45.09 *x* − 0.30		0.9999	0.04		0.13
**Analyte**	**Precision as RSD (%)**		**Accuracy as Recovery ± RSD (%)**					
	**Intra-Day Precision** **(*n* = 6)**	**Inter-Day** **Precision** **(*n* = 9)**	**Low** **(*n* = 3)**	**Medium** **(*n* = 3)**			**High** **(*n* = 3)**	
SASP	0.08	0.10	99.14 ± 0.16	99.74 ± 0.09			99.27 ± 0.16	
FA	0.77	0.78	101.71 ± 2.05	100.45 ± 0.96			101.54 ± 0.76	
SA	0.35	0.35	103.84 ± 0.76	102.50 ± 0.37			102.25 ± 0.46	
SP	0.67	0.67	101.15 ± 1.40	101.01 ± 0.34			99.59 ± 1.01	

**Table 2:** Eigenvalues and variances of the nine obtained principal components.

Eigenvector	1	2	3	4	5	6	7	8	9
Eigenvalue	494.6	134.8	7.6	6.2	2.4	1.9	1.0	0.4	0.2
Variability (%)	76.21	20.77	1.17	0.95	0.37	0.29	0.15	0.07	0.03
Cumulative (%)	76.21	96.98	98.15	99.10	99.47	99.76	99.91	99.97	100.00

**Table 3:** Results of the chromatographic analysis after isothermal stress testing (IST).

API	Sample Type	Appearance	Physical Change	Recovery ± RSD (%) (*n* = 3)
SASP	standard	brownish-yellow powder	no significant visual changes	99.0 ± 0.5
	tablet			102.5 ± 1.3
	standards blend			99.9 ± 2.1
	tablets blend			101.0 ± 1.2
FA	standard	yellow powder	no significant visual changes	98.4 ± 0.6
	tablet	pale yellow powder		99.3 ± 1.0
	standards blend	brownish-yellow powder		98.6 ± 1.6
	tablets blend	brownish-yellow powder		± 2.7

**Table 4:** Forced degradation study results.

Degradation Condition	Degradation Times/day ^1^	Observed Drops of Assay/%					
		Standards		Standards Blend		Tablets Blend	
		SASP	FA	SASP	FA	SASP	FA
0.1 M NaOH (solution)	7	1.8	2.7	0.8	2.7	2.3	2.4
0.1 M HCl (solution)	7 (1)	0.8	21.6	0.4	17.4	1.7	19.0
3% H_2_O_2_ (solution)	7 (1)	1.7	11.7	n.d. ^2^	11.8	2.2	10.4
Photo (solution)	7 (1)	1.5	10.0	0.3	14.6	1.6	16.7
Photo (solid)	7	1.7	2.9	1.3	0.6	1.6	3.7
Thermal (solution)	7	1.9	1.1	n.d.	100.0	n.d.	100.0
Thermal (solid)	7	1.9	1.0	2.4	1.8	0.2	2.2

^1^ Values in the parentheses represent the optimal degradation time for the FA standard and FA in the prepared blends. ^2^ n.d. = no degradation observed.

## Data Availability

The data presented in this study are available on request from the corresponding author. The data are not publicly available due to privacy or ethical restrictions.
